# Next‐generation sequencing in pediatric‐onset epilepsies: Analysis with target panels and personalized therapeutic approach

**DOI:** 10.1002/epi4.13039

**Published:** 2024-08-31

**Authors:** Barbara Castellotti, Francesca Ragona, Elena Freri, Giuliana Messina, Stefania Magri, Roberto Previtali, Roberta Solazzi, Silvana Franceschetti, Franco Taroni, Laura Canafoglia, Cinzia Gellera, Tiziana Granata, Jacopo C. DiFrancesco

**Affiliations:** ^1^ Unit of Medical Genetics and Neurogenetics Fondazione IRCCS Istituto Neurologico Carlo Besta Milan Italy; ^2^ Department of Pediatric Neuroscience Fondazione IRCCS Istituto Neurologico Carlo Besta Milan Italy; ^3^ Pediatric Neurology, Department of Biomedical and Clinical Sciences, Buzzi Children's Hospital University of Milan Milan Italy; ^4^ Integrated Diagnostics for Epilepsy Fondazione IRCCS Istituto Neurologico Carlo Besta Milan Italy; ^5^ Department of Neurology Fondazione IRCCS San Gerardo Dei Tintori Monza Italy

**Keywords:** next‐generation sequencing, panels of analysis, pediatric epilepsy, personalized medicine

## Abstract

**Objective:**

The objective of this study is to report the results of the genetic analysis in a large and well‐characterized population with pediatric‐onset epilepsies and to identify those who could benefit from precision medicine treatments.

**Methods:**

In this retrospective observational study, we consecutively recruited patients with pediatric‐onset epilepsy observed at a tertiary neurological center over a time span of 7 years, collecting clinical and laboratory findings. Following in‐depth diagnostic process to exclude possible structural and metabolic causes of the disease, patients with a suspected genetically determined etiology underwent next‐generation sequencing (NGS) screening with panels for the analysis of target genes causative of epilepsy.

**Results:**

We detected likely pathogenic or pathogenic variants (classes IV and V) in 24% of the 562 patients who underwent genetic investigations. By the evaluation of patients' data, we observed that some features (onset of epilepsy before one year old, presence of neurological deficits, psychomotor delay/cognitive disability, and malformative aspects at brain MRI) were significantly associated with class IV or V variants. Moreover, statistical analysis showed that the diagnostic yield resulted higher for patients affected by Progressive Myoclonic Epilepsy (PME) and with early onset developmental and epileptic encephalopathies (DEE), compared with focal epilepsies, genetic generalized epilepsies, DEE with onset at/after 1 y.o., and unclassified epileptic syndromes. According to the results of the genetic screening, up to 33% of patients carrying class IV or V variants resulted potentially eligible for precision medicine treatments.

**Significance:**

The large‐scale application of NGS multigene panels of analysis is a useful tool for the molecular diagnosis of patients with pediatric‐onset epilepsies, allowing the identification of those who could benefit from a personalized therapeutic approach.

**Plain Language Summary:**

The analysis of patients with pediatric‐onset epilepsy using advanced technologies for the screening of all the implicated genes allows the identification of the cause of diseases in an ever‐increasing number of cases. Understanding the pathogenic mechanisms could, in some cases, guide the selection and optimization of appropriate treatment approaches for patients.

**Key points**

- Next‐generation sequencing analysis with multigene panels is a valid approach in the diagnostic process of pediatric‐onset epilepsies.
- Phenotypic features including onset of epilepsy before one year old, neurological deficits, psychomotor delay/cognitive disability, and malformative aspects at brain MRI are significantly associated with class IV or V variants.
- Diagnostic rate is higher in patients with PME and early‐onset DEEs.
- One third of patients carrying pathogenic or likely pathogenic variants could be eligible for a personalized therapeutic approach.

## 1 INTRODUCTION

The large‐scale application of next‐generation sequencing (NGS) techniques has improved our knowledge of the complex pathogenic mechanisms underlying pediatric‐onset epilepsies, particularly those featuring as developmental and epileptic encephalopathies (DEEs). A precise molecular diagnosis is essential for patients and their families, providing a definitive explanation for the disease. This also enables a more targeted genetic counseling, including information about the prognosis of affected individuals and the risk of recurrence for any future pregnancy.^1^ A conclusive diagnostic result also holds significant psychological implications, preventing the “*diagnostic Odyssey*”, the wandering of patients and their families in search of a diagnosis.^2^

Moreover, understanding the pathogenic mechanisms of the disease offers the opportunity for the application of precision medicine procedures, defined as any treatment applied on the basis of the patient's specific genetic cause.^3^, ^4^, ^5^, ^6^ This is particularly relevant considering that 25%–33% of patients with monogenic epilepsy can benefit of a potential precision medicine approach,^7^, ^8^ resulting in a substantial reduction of side effects related to pharmacological polytherapies, and significant economic savings for the health system, mainly in term of treatment and hospitalization.

According to the knowledge available in the literature, we can consider different levels of efficacy of personalized medicine treatments.^6^, ^9^, ^10^, ^11^ An *established* approach lays on solid data obtained through randomized clinical trials. A *potential* treatment is based on small or non‐randomized experiences, and should be considered on an individual basis, after the careful assessment of the risks/benefits for the patient, and, ideally, supported by functional studies for the in vitro evaluation of the response to the proposed treatment.^12^, ^13^ Finally, approaches only based on the experimental data can be considered as *hypothetical*.

Herein, we report the results of the genetic screening with target panels in a large and well‐characterized population of patients with pediatric‐onset epilepsies, observed at a highly specialized Italian Centre for the diagnosis and cure of neurological diseases.

## 2 METHODS

### 2.1 Ethical approval and patients' consent

This study received the ethical approval by the institutional review boards of the Fondazione IRCCS Istituto Neurologico Carlo Besta of Milan, Italy. Written informed consent for genetic analysis and use of anonymized clinical data for research purposes was obtained from all patients or guardians of participants. This research was performed in accordance with GCP and the ethical standards laid down in the 1964 Declaration of Helsinki.

### 2.2 Patients' recruitment

In this retrospective observational study, we evaluated the results of NGS multigene analysis in patients with pediatric‐onset epilepsy with suspected genetically determined etiology consecutively observed at the Fondazione IRCCS Istituto Neurologico Carlo Besta of Milan. Patient's recruitment and genetic screening were assessed from January 2014 to December 2021; diagnosis of epilepsy was established according to the most recent criteria from the International League Against Epilepsy (ILAE).^14^, ^15^, ^16^

To exclude those with an acquired structural brain damage of non‐genetic origin (for example cerebrovascular, hypoxic, tumoral), and metabolic causes of epilepsy, patients underwent to an in‐depth diagnostic process which encompassed extensive clinical and laboratory evaluations, including: neurophysiological studies (video EEG in wakefulness and sleep, polygraphy, evoked potential), neuropsychological assessments (developmental milestones, cognitive function, and behavior), brain MRI imaging (1.5 or 3T), and, in selected cases, screening for neurometabolic disorders. Preliminary genetic assessment included CGH array and karyotyping. Single gene sequencing and Multiplex dependent Legation Probe Amplification (MLPA) were considered for those whose phenotype suggested a specific etiology: *SCN1A* (Dravet syndrome), *PCDH19*, *SLC2A1* (Glut1 deficiency syndrome), *PRRT2*, *FLNA* (periventricular heterotopia), *EPM2A* and *NHLRCA1* (Lafora disease).

### 2.3 Genetic screening by NGS target panels

Inclusion criteria for the genetic analyses were as follows: study consent provided by caregivers, confirmed diagnosis of epilepsy with suspected genetically determined etiology, DNA availability of both parents (and other adjunctive relatives when requested, e.g. affected siblings) for segregation analysis of identified variants.

Genomic DNA was extracted from peripheral blood lymphocytes, according to standard procedures. Overtime, we designed target panels using the Illumina design studio software (https://designstudio.illumina.com) for panels containing up to 100 genes and Agilent's SureDesign tool (https://earray.chem.agilent.com/suredesign/) for the panels containing more than 100 genes. All the panels included genes specifically selected due to their involvement in the pathogenesis of infantile and childhood epilepsies. These panels cover exonic regions and exon±intron boundaries of selected genes. We designed a total of eight different target panels, each single patient was analyzed at least with one of the panels performed. The list of the analyzed genes is reported in Figure S1.

Enriched libraries were sequenced with MiSeq apparatus (Illumina). Raw data were analyzed by a pipeline using both online available softwares and customized internal protocols. The resulting sequences were aligned to the reference genome (GRCh37/hg19) using the MiSeq software. Coverage with a minimum reading depth of 20X for each nucleotide was found to be above 99%. The regions of genes considered particularly interesting and found to have insufficient coverage, were subsequently sequenced by direct Sanger sequencing.

The data analysis was obtained using the following software: Illumina MiSeq Reporter vs 2.4.60, Illumina Variant Studio vs 2.2, Qiagen CLC Genomics Workbench vs 7.0. Variants with MAF >1% reported in the dbSNP (https://www.ncbi.nlm.nih.gov/projects/SNP/), 1000 Genome (browser.1000genomes.org), EVS database (evs.gs.washington.edu), ExAC database (http://exac.broadinstitute.org/) and Varsome (https://varsome.com/) were considered benign variants and excluded from the report. The “in silico” simulation for possibly splicing mutations was evaluated by Alternative Splice Site Prediction (http://wangcomputing.com/assp/) or Human Splicing Finder (http://www.umd.be/HSF/HSF.shtml).

The genetic variants passing the filtering process were classified as “pathogenic” (class V), “likely pathogenic” (class IV), “variants of unknown significance” (VoUS, class III), “unlikely pathogenic” and “not pathogenic variants” (class II–class I), according to ACMG criteria.^17^ In case of identification of genetic variants class III to V, we performed segregation analysis by Sanger sequencing of both parents and, if necessary, the siblings (affected and/or healthy). Following NGS genetic screening, each case was collectively discussed between geneticists and referring clinicians, for the evaluation of the possible variant(s) identified, based on the phenotypic characteristics of the patient.

### 2.4 Clinical data collection

We extracted medical records from a pseudo‐anonymized database (available exclusively to the study researchers) the following information: gender, age of epilepsy onset, family history of epilepsy, geographical origin; neurological features (pathological neurological examination, psychomotor retardation/cognitive impairment); neuroradiological data (malformative aspects at brain MRI); neurophysiological data (epileptiform activity at EEG). We also collected the following epileptic features: epileptic syndrome, seizure control on anti‐seizure medications (ASMs), seizure freedom after discontinuation of ASMs, and drug‐resistant epilepsy.

### 2.5 Statistical analysis

To identify any difference in the phenotypic characteristics of patients with (genetically *solved*) and without (*unsolved*) pathogenic variants, we compared clinical and instrumental data of the two categories of subjects. Quantitative statistics were analyzed using the R software (www.r‐project.org). The statistical significance was set as *p* < 0.05. *χ* ^2^ analyses were used to explore differences on clinical and instrumental features between genetically solved and unsolved patients. Diagnostic rate was calculated as the number of solved patients per category (type of epileptic syndrome) and expressed as percentage of the total number of patients in the corresponding category. *χ* ^2^ test were used to examine overall association of type of epileptic syndrome with diagnostic rate. Standardized residuals were used to identify the most contributing categories, the contribution (expressed as %) of a given category to the total *χ* ^2^ score is calculated as the ratio between each squared residuals and *χ* ^2^.

## 3 RESULTS

### 3.1 NGS genetic screening

We consecutively recruited 562 patients with pediatric‐onset epilepsy of suspected genetic etiology who underwent genetic screening by NGS target panels. Of these, we retrieved clinical‐instrumental data of 488, whereas information was missing for the remaining 74 subjects.

A molecular diagnosis was achieved in 135 of the 562 patients, with an overall yield of 24%. We identified class V variants in 75 patients (13%) and class IV in 60 (11%). Moreover, the genetic analysis reported VoUS (class III) in 25% and benign or likely benign variants (classes I and II) in the remaining 51% (Table 1).

**TABLE 1:** Results of NGS genetic screening.

Class of variants	Number of patients	%	Grouped results	Grouped %
5	75	13	135	24
4	60	11		
3	140	25	427	76
1 or 2	287	51		
Total	562	100		

*Note*: Classification of variants identified in patients with pediatric‐onset epilepsy of suspected genetic etiology analyzed with epilepsy target panels. Values are expressed as number of patients tested and percentage.

### 3.2 Characteristics of the study population

The main characteristics of the 562 patients included in the NGS genetic screening are summarized in Table 2. In this population, we observed a similar gender distribution (female 48%); 22% of patients experienced onset of epilepsy within the age of 1 y.o., 33% had a positive family history for epilepsy and 86% were of European origin. Neurological deficits were present in 37% of cases, and psychomotor delay/cognitive deficit in 57%. Brain MRI detected malformations in 43% of patients. Epileptiform activity at the EEG was identified in 66%. Control of seizures was reached in 41% of those treated with ASMs, whereas only 9% obtained seizure freedom at discontinuation of ASMs; epilepsy was refractory to ASMs in 32% of cases.

**TABLE 2:** Phenotypic characteristics of the study population.

Characteristics of patients	Total number of patients	Patients with pathogenic variants	Patients without pathogenic variants	*p*‐Value solved (class IV–V) versus unsolved (class I–III)
Gender (F/M)	271/291	70/65	201/226	0.3844
Age of epilepsy onset (</≥1 year/U)	125/327/110	47/74/14	78/253/96	**0.001958**
Family history for epilepsy (N/Y/U)	291/187/84	84/43/8	207/144/76	0.1895
Geographical origin (EUR/NON EUR)	486/76	117/18	369/58	1
Altered neurological examination (N/Y/U)	268/209/85	59/72/4	209/137/81	**0.00355**
Psychomotor delay/cognitive defect (N/Y/U)	157/320/85	32/99/4	125/221/81	**0.02045**
Malformative brain MRI (N/Y/U)	210/242/110	69/55/11	141/187/99	**0.02135**
EEG epileptiform activity (N/Y/U)	72/370/120	14/99/22	58/271/98	0.2486
Seizure control with ASMs (N/Y/U)	174/231/157	48/55/32	126/176/125	0.454
Seizure freedom without ASMs (N/Y/U)	414/49/99	110/8/17	304/41/82	0.1668
Drug resistance (N/Y/U)	253/180/129	58/57/20	195/123/109	0.05492

*Note*: Values are expressed as number of patients for the phenotypic characteristic considered (N = no, Y = yes, U = unknown); *p*‐values are calculated by the comparison between genetically solved and unsolved patients. The statistical significance was set as *p* < 0.05. Significant values are reported in bold.

Statistical analysis demonstrated that the following phenotypic features were significantly associated with the presence of class IV or V variants: onset of epilepsy within the first year of life, presence of neurological deficits, psychomotor delay/cognitive defect, and abnormalities at brain MRI. Significant values are reported in bold in Table 2.

### 3.3 Epileptic syndromes and diagnostic rate

We classified recruited patients into the following epileptic syndromes: early onset DEE (including early infantile DEE, infantile epileptic spasm syndrome, and Dravet syndrome, 83 patients); structural focal epilepsies (57); other focal epilepsies (86); genetic generalized epilepsies (including myoclonic epilepsy in infancy, generalized epilepsy with febrile seizures+, epilepsy with myoclonic‐atonic seizures, epilepsy with myoclonic absences, childhood absence epilepsy, epilepsy with myoclonic absences, juvenile absence epilepsy, juvenile myoclonic epilepsy, epilepsy with generalized tonic–clonic seizures, 169); DEE with onset at/after 1 y.o. (including Lennox–Gastaut syndrome, epileptic encephalopathy with spike–wave activation in sleep, DEE with spike–wave activation in sleep, undefined DEE with onset at/after 1 y.o., 46); Progressive Myoclonic Epilepsy (PME, 37); unclassified epileptic syndromes (84).

We observed a significantly higher diagnostic rate of NGS genetic analysis in patients affected by PME (16/37 patients) and with early onset DEE (29/83), compared with other focal epilepsies (25/86, 29%), structural focal epilepsies (15/57, 26%), genetic generalized epilepsies (33/169, 20%), DEE with onset at/after 1 y.o. (7/46, 15%), and unclassified epileptic syndromes (10/84, 12%). Significant values are reported in bold in Table 3.

**TABLE 3:** Diagnostic rate of NGS target panels across epileptic syndromes.

Epileptic syndrome	Total number of patients	Patients with pathogenic variants (solved)	Diagnostic rate (%)	Solved and unsolved standardized residuals (contribution as %)
Early onset DEE	83	29	35	**2.030** (16.6%) −1.141 (5.2%)
Structural focal epilepsies	57	15	26	0.353 (0.5%) −0.199 (0.2%)
Other focal epilepsies	86	25	29	0.955 (3.7%) −0.537 (1.2%)
Genetic generalized epilepsies	169	33	20	−1.192 (5.7%) 0.670 (1.8%)
DEE with onset at/after 1 y.o.	46	7	15	−1.218 (6.0%) 0.685 (0.7%)
Progressive Myoclonic Epilepsy (PME)	37	16	43	**2.386** (22.9%) −1.341 (7.2%)
Unclassified epileptic syndromes	84	10	12	**−2.266** (20.7%) 1.274 (6.5%)
Total	562	135	24	*p*‐Value = 0.0003627

*Note*: Values are expressed as the number of patients with a specific epileptic syndrome, those with a pathogenic variant identified by the NGS genetic screening (solved), and the relative diagnostic rate (expressed as percentage) for each epileptic syndrome. *χ* ^2^ test identify an overall association of type of epileptic syndrome with diagnostic rate (*χ* ^2^ = 24.858, df = 6, *p*‐value = 0.0003627). Standardized residuals were used to identify the most contributing categories, the contribution (%) of a given category to the total *χ* ^2^ score is calculated as the ratio between each squared residuals and *χ* ^2^. Significant values are reported in bold.

### 3.4 Personalized therapeutic approach

**FIGURE 1:**
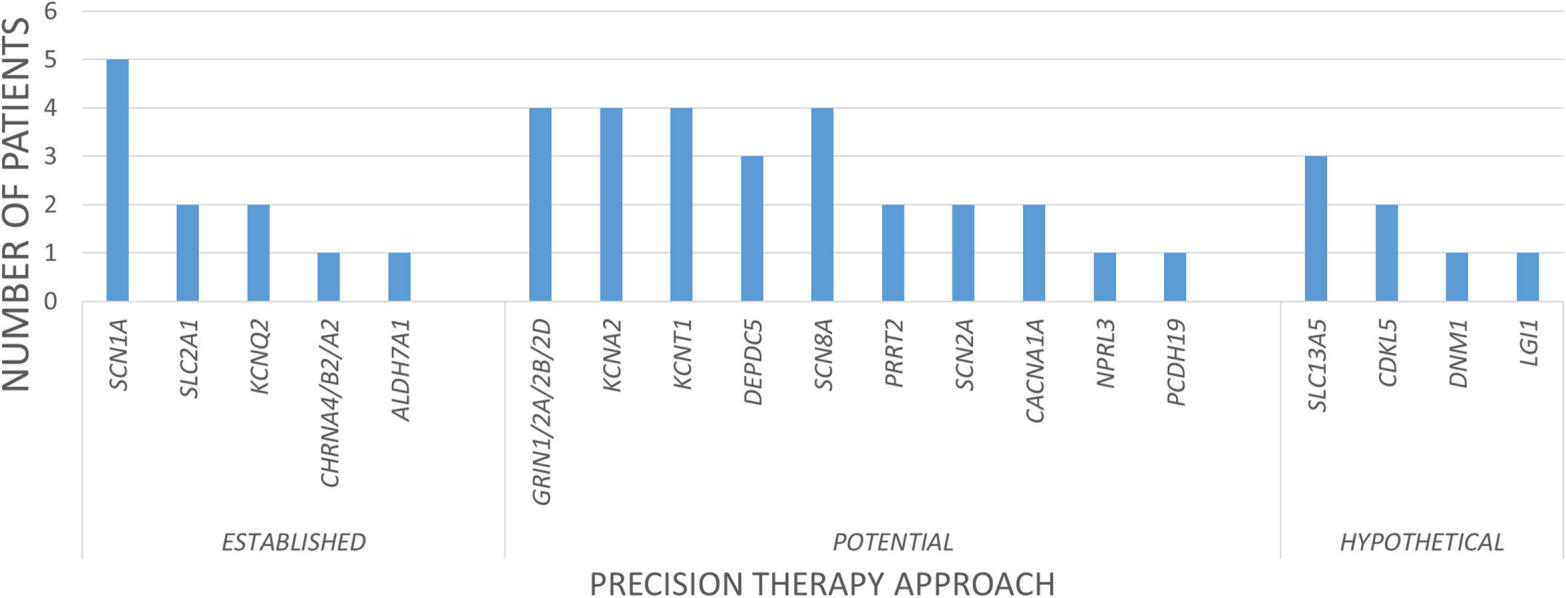
Patients with pathogenic variants eligible for personalized therapeutic approaches. Values are reported as number of patients carrying pathogenic variants for a single gene, grouped based on the different treatment opportunities (*established*, *potential*, and *hypothetical*).

Among the 135 patients with solved genetic diagnosis, we identified 45 (33%) eligible for a personalized therapeutic approach (Figure 1). Based on the current scientific knowledge, 11 (24%) resulted with pathogenic variants in genes for which specific treatments are *established*: *SCN1A* (5 cases), *SLC2A1* (2), *KCNQ2* (2), *CHRNA4* (1), *ALDH7A1* (1). The largest group was represented by patients carrying variants in genes for which personalized treatments are *potential* (27/45, 60%): *GRIN1/2A/2B/2D* (4 patients), *KCNA2* (4), *KCNT1* (4), *DEPDC5* (3), *SCN8A* (4), *PRRT2* (2), *SCN2A* (2), *CACNA1A* (2), *NPRL3* (1), and *PCDH19* (1). In the remaining 7 patients (16%), we identified causative variants in genes for which there are *hypothetical* possibilities for a specific treatment: *SLC13A5* (3 patients), *CDKL5* (2), *DNM1* (1), and *LGI1* (1).

## 4 DISCUSSION

In this retrospective observational study, we report the genetic screening of a large and well‐characterized population of pediatric‐onset epilepsies, identifying those cases who could benefit from precision medicine treatments. The application of NGS technology through panels for the sequencing of genes implicated in the pathogenesis of epilepsies brought to the molecular diagnosis in 135 of the 562 subjects recruited, with a diagnostic yield of 24%, in line with comparable previously reported case series.^18^, ^19^, ^20^, ^21^

Since the multigene panels applied in this study demonstrated a high coverage of all the coding sequences for the genes analyzed (nearly 100%), we could exclude any missing variants of potential diagnostic interest in the coding and splicing regions. Through the application of target panels, we verified the presence of copy number variants, especially in genes with an autosomal recessive transmission, in the presence of a single heterozygous variant of possible pathogenic significance. The segregation in parents and siblings of variants identified in classes III‐IV‐V and the collegial discussion of each individual case between clinicians and geneticists led to the best interpretation of the genetic results. Moreover, thanks to the selective screening of target genes for epilepsy, we avoided the identification of potential incidental findings that might be difficult to manage, especially in a pediatric population.^22^

However, despite the screening of target genes for pediatric epilepsies, a high percentage of patients remained without a molecular diagnosis. While in 25% we identified VoUS (class III) that could theoretically play a pathogenic role, although not yet clarified,^23^ in more than half of the subjects (51%) no variants of potential interest were reported. These cases could benefit from a more broad‐spectrum genetic screening, as whole exome or genome sequencing (WES or WGS).^6^, ^24^, ^25^

In line with previous experience,^26^ we obtained a molecular diagnosis more frequently in patients with definite phenotypic features, characterized by early onset epilepsy, presence of neurological deficits, developmental delay, and abnormalities at brain MRI. Moreover, the diagnostic rate of target panes resulted higher for patients affected by PME and early onset DEE, compared with other epileptic syndromes. These data suggest the opportunity to quickly refer patients with these characteristics to NGS analysis, to avoid further delays in the diagnosis and procrastinating an unnecessary diagnostic Odyssey.

The identification of a correct molecular diagnosis in genetically determined diseases is fundamental for the application of personalized medicine strategies,^3^, ^4^, ^5^, ^6^ that can significantly improve patients' outcome.^27^ Thanks to the constant improvement and diffusion of NGS genetic investigation techniques, a personalized medicine approach is increasingly available for difficult‐to‐treat monogenic epilepsies.^26^ To date, there are several examples of established personalized therapies based on the altered function caused by pathogenic variants. This approach can, for example, be obtained through the application of ASMs with specific functions, as the sodium channel blockers for the treatment of gain‐of‐function (GoF) variants in *SCN2A* and *SCN8A*,^28^ but also with non‐ASM compounds, as the mammalian target of rapamycin (mTOR) inhibitors for mTORopathies.^29^ In this scenario, non‐pharmacological approaches are also included, as the ketogenic diet (KD) for the treatment of patients with Glut1 deficiency syndrome.^30^, ^31^ Moreover, the repurposing of already available compounds, not necessarily with anti‐seizure function, but indicated for different conditions, can also be effective for the treatment of severe monogenic epilepsies.^32^, ^33^, ^34^, ^35^

The genetic screening of the large cohort herein analyzed, demonstrated that more than one third of the patients who reached a molecular diagnosis could benefit from precision medicine treatments. While for some pathogenic variants the possibilities of a precision treatment are currently only hypothetical or potential and require further investigation to be applicable on a large scale, for others there is concrete evidence supporting their application in the clinic.

## 5 LIMITATIONS

This study has some limitations. Being retrospective, for genetic analyses we included results obtained by different panels with increasing numbers of genes screened. According to the growing knowledge of the pathogenic mechanisms of epilepsy, the number of genes associated with the disease is constantly expanding. To partially overcome this limitation, we recruited a large cohort of patients.

As expected, there is a prevalence of patients of European origin, Italians in particular. Thus, our genetic results may be partly affected by the specific characteristics of the population analyzed. However, these data overlap with previous experiences in non‐Italian populations.^18^, ^19^, ^20^, ^21^

Another limitation can be considered the extreme phenotypic variability of the patients recruited, making the analyzed cohort heterogeneous. Moreover, the Fondazione IRCCS Istituto Neurologico Carlo Besta of Milan is a reference center for pediatric‐onset epilepsy in Italy, and some patients included in this study came from external centers as a second observation. This therefore implies a possible selection bias of more complex cases. However, the high sample size included in this study allowed the evaluation of different phenotypes of childhood‐onset epilepsy, making the study more informative.

A final consideration regards precision therapies which must always be considered case‐by‐case. First, for reasons yet to be clarified, some patients carrying pathogenic variants potentially deserving of precision treatment may not respond to targeted therapies.^36^, ^37^, ^38^ Second, not all subjects with epilepsy caused by variants potentially treatable with precision medicine need to change their treatments in favor of more specific ones. Some of these subjects are already under satisfactory seizure control with conventional ASMs, selected based on clinical and instrumental characteristics. However, if in the future there would be a worsening evolution of the clinical picture, mainly due to the loss of efficacy of traditional therapies, knowing the molecular cause of the disease could allow the rapid activation of more targeted treatments.

## 6 CONCLUSIONS

The large‐scale application of NGS genetic analysis using multigene panels in patients with pediatric‐onset epilepsies allowed the identification of the molecular diagnosis in approximately a quarter of cases, where the diagnostic rate is higher for those with PME and early onset DEEs. Our results confirm that these techniques are recommended in particular for those with specific phenotypic characteristics, i.e. onset of epilepsy before 1 y.o., altered neurological examination, psychomotor delay/cognitive defect and malformative brain MRI. The knowledge obtained through molecular diagnosis gives the opportunity to identify patients who could be treated using precision medicine approaches.

## 7 CLINICAL RELEVANCE

Any further advance in the diagnostic accuracy of pediatric patients with epilepsy could lead to a better definition of the pathogenic mechanisms of the disease, helping to identify new personalized treatment strategies for affected individuals.

## AUTHOR CONTRIBUTIONS

Patients' recruitment, characterization and data collection (RP, RS, FR, EF, LC, TG), genetic analysis (BC, GM, SM, FT, CG), statistical analysis (SM), study planning and coordination (JCD, BC, SM, TG, CG), writing of manuscript (JCD, BC).

## FUNDING INFORMATION

The present work was supported by the Italian Ministry of Health Project Ricerca Finalizzata Giovani Ricercatori GR‐2016‐02363337 to JCD and SM, and Project Ricerca Finalizzata RF‐2019‐12 370 491 to BC.

## CONFLICT OF INTEREST STATEMENT

The authors declare that the research was conducted in the absence of any commercial or financial relationships that could be construed as a potential conflict of interest.

## ETHICS STATEMENT

We confirm that we have read the Journal's position on issues involved in ethical publication and affirm that this report is consistent with those guidelines.

## Supporting information


Figure S1.
