# Supplementary figures and images for: Next‐generation sequencing in pediatric‐onset epilepsies: Analysis with target panels and personalized therapeutic approach
Source: Epilepsia Open. 2024 Aug 31;9(5):1922–30. doi: 10.1002/epi4.13039 (PMC11450606; doi:10.1002/epi4.13039)

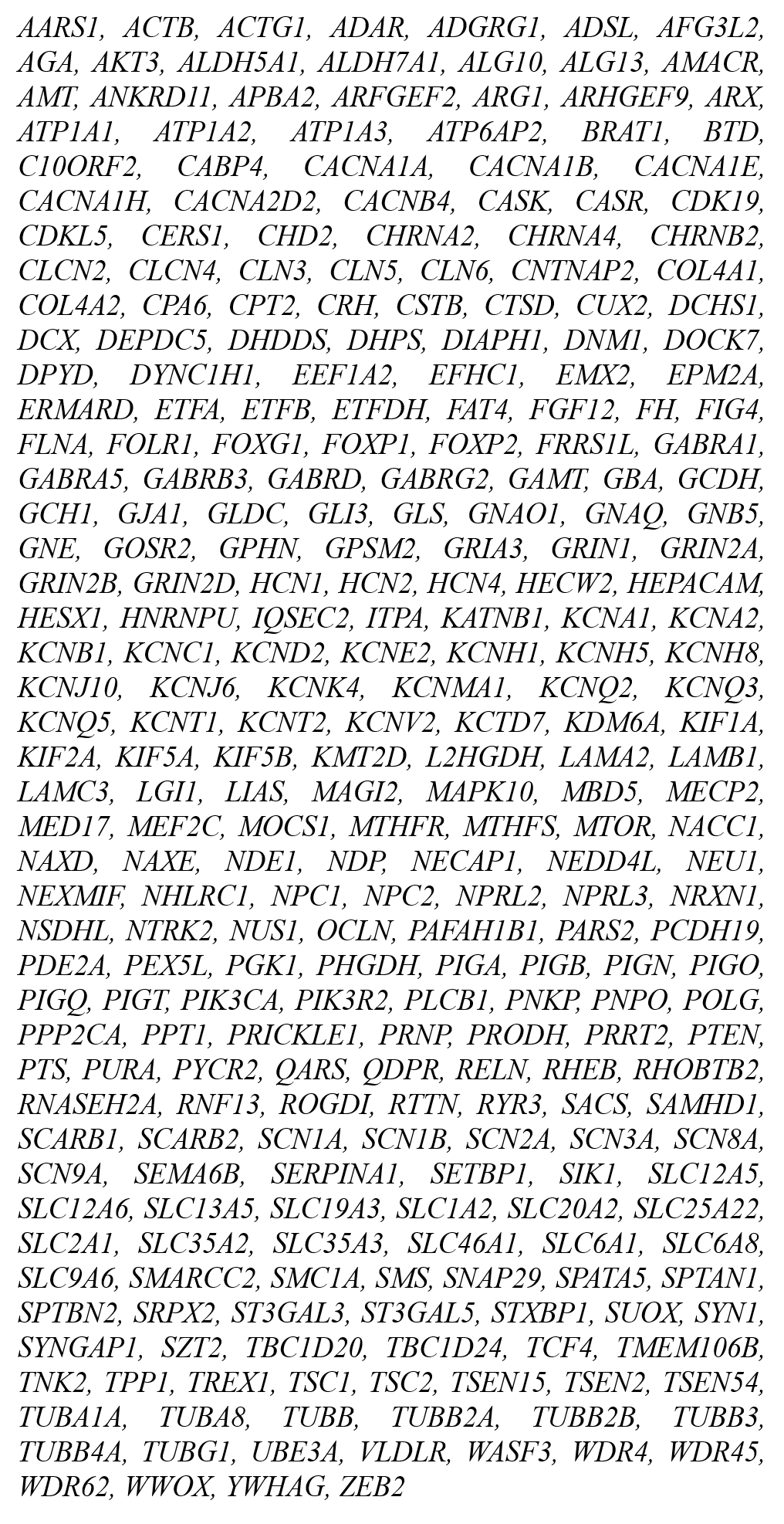

Supplement: Supplementary file 1 — Figure S1. [file EPI4-9-1922-s001.tif]
